# Dihydroxy-Metabolites of Dihomo-γ-linolenic Acid Drive Ferroptosis-Mediated Neurodegeneration

**DOI:** 10.1021/acscentsci.3c00052

**Published:** 2023-03-16

**Authors:** Morteza Sarparast, Elham Pourmand, Jennifer Hinman, Derek Vonarx, Tommy Reason, Fan Zhang, Shreya Paithankar, Bin Chen, Babak Borhan, Jennifer L. Watts, Jamie Alan, Kin Sing Stephen Lee

**Affiliations:** † Department of Chemistry, Michigan State University, East Lansing, Michigan 48824, United States; ‡ Department of Pharmacology and Toxicology, Michigan State University, East Lansing, Michigan 48824, United States; § School of Molecular Biosciences, Washington State University, Pullman, Washington 99164, United States; ∥ Department of Pediatrics and Human Development, Michigan State University, Grand Rapids, Michigan 49503, United States

## Abstract

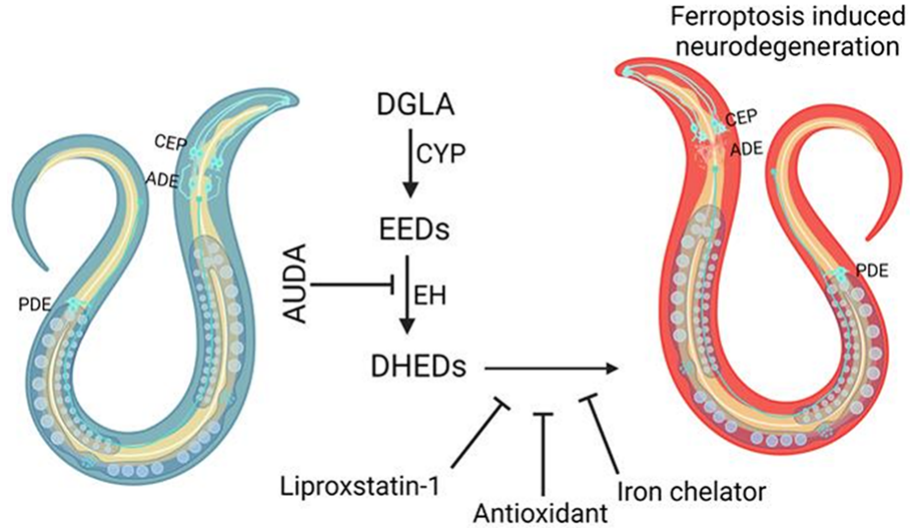

Even after decades of research, the mechanism of neurodegeneration remains understudied, hindering the discovery of effective treatments for neurodegenerative diseases. Recent reports suggest that ferroptosis could be a novel therapeutic target for neurodegenerative diseases. While polyunsaturated fatty acid (PUFA) plays an important role in neurodegeneration and ferroptosis, how PUFAs may trigger these processes remains largely unknown. PUFA metabolites from cytochrome P450 and epoxide hydrolase metabolic pathways may modulate neurodegeneration. Here, we test the hypothesis that specific PUFAs regulate neurodegeneration through the action of their downstream metabolites by affecting ferroptosis. We find that the PUFA dihomo-γ-linolenic acid (DGLA) specifically induces ferroptosis-mediated neurodegeneration in dopaminergic neurons. Using synthetic chemical probes, targeted metabolomics, and genetic mutants, we show that DGLA triggers neurodegeneration upon conversion to dihydroxyeicosadienoic acid through the action of CYP-EH (CYP, cytochrome P450; EH, epoxide hydrolase), representing a new class of lipid metabolites that induce neurodegeneration via ferroptosis.

## Abstract 2

The ω-6 dihomo-γ-linolenic acid (DGLA) triggers ferroptosis-mediated neurodegeneration through its downstream cytochrome P450-epoxide hydrolase (CYP-EH) metabolites, DHED.

## Introduction

By 2050, the projected population older than age 65 is expected to be more than double, reaching over 1.5 billion, and the projected population older than 80 is predicted to triple to 426 million.^1^ As aging is a risk factor for neurodegeneration, it is expected that the population with dementia will significantly increase in the near future.^2^ However, the mechanisms of neurodegeneration remain unclear, and effective preventative measures and treatment are currently lacking.^3^ Therefore, identifying the molecular mechanisms underlying neurodegeneration is an unmet medical need. While tauopathy, neuroinflammation, and excitotoxicity may play key roles in neurodegeneration, recent studies provide compelling evidence that ferroptosis could be a new mechanism underlying neurodegeneration.^3−5^ Ferroptosis is a non-apoptotic form of regulated cell death that is driven by an increase of iron-dependent lipid peroxidation in the cellular membrane.^4,6^ Epidemiological studies showed that patients with Parkinson’s disease (PD) or Alzheimer’s disease (AD) have elevated iron and lipid peroxide levels in the brain compared to healthy controls, which is consistent with ferroptosis.^5,7−12^ The regulatory mechanism of ferroptosis in brain cells is understudied, although polyunsaturated fatty acids (PUFAs) play a critical role in this process.^13−17^

PUFAs are key structural components of plasma membranes and play a critical role in neuronal functions.^18^ Generally, ω-3 and ω-6 PUFAs are two of the major classes of PUFAs present in the human diet.^19^ Human studies have demonstrated that an increase in the plasma ω-3/ω-6 PUFA ratio decreases the risk of neurodegenerative diseases, including AD and PD.^20−23^ Nonetheless, even after decades of epidemiological studies in mammalian and cell-based models, how PUFAs affect neurodegeneration is poorly understood with reported results that are contradictory.^21,24,25^ While most efforts in research have investigated the neuroprotective effects of ω-3 PUFA supplementation, few studies have examined the role of ω-6 PUFAs in neurodegeneration.^26−28^ This is surprising since the modern western diet has dramatically increased our consumption of ω-6 PUFAs.^29,30^ While the exact role of ω-6 PUFAs in neurodegenerative diseases is not understood, it is known that supplementing mammalian cells with ω-6 PUFAs sensitizes cells to ferroptosis.^13−15,31^ In addition, ω-6 dihomo-γ-linolenic acid (20:3n-6, DGLA) induces ferroptosis in the *Caenorhabditis elegans* germline, while earlier studies have suggested that an epoxide metabolite of DGLA may mediate germ cell death.^17,32^

Although the mechanisms by which ω-6 PUFAs mediate biological effects remain undefined, recent studies have demonstrated that ω-6 PUFA metabolites resulting from cytochrome P450 (CYP) enzymes and epoxide hydrolases (EHs) action are key signaling molecules for human physiology.^18,33,34^ As such, this study was initiated to test whether specific ω-6 PUFAs modulate neurodegeneration via their downstream CYP metabolites and to investigate whether ferroptosis plays a role in the observed biology. Because the CYP enzymes and EHs are differentially expressed in tissues and cell types (Table S1),^35−39^ and the expressions of both enzymes are significantly affected by cell passages,^40−42^ it is difficult to pinpoint a specific cell line suitable for our study. Therefore, a whole animal study is necessary to uncover this novel mechanism without worrying about metabolites not being generated locally or overlooking critical cell–cell communications facilitated by these lipid metabolites.

To facilitate our study, we took an interdisciplinary approach by combining a simple genetic animal model, an inhibitor of a metabolic enzyme, synthesized lipid metabolites, and targeted metabolomics to systematically investigate the crosstalk among lipid metabolism, neurodegeneration, and ferroptosis. With this approach, we first demonstrate that among five tested PUFAs, only DGLA induces neurodegeneration in select neurons in *C. elegans*, with more pronounced effects in dopaminergic neurons, and to a lesser extent in glutaminergic neurons, with no observable effects in cholinergic and GABAergic neurons. Furthermore, we demonstrate that the DGLA-induced neurodegeneration is mediated through its downstream CYP-EH metabolite, dihydroxyeicosadienoic acid (DHED), and ferroptosis is likely the mechanism involved in DHED-induced neurodegeneration.

## Results

### DGLA, but Neither ω-3 nor Other ω-6 PUFAs, Induces Degeneration Specifically in Dopaminergic Neurons

**Figure 1:**
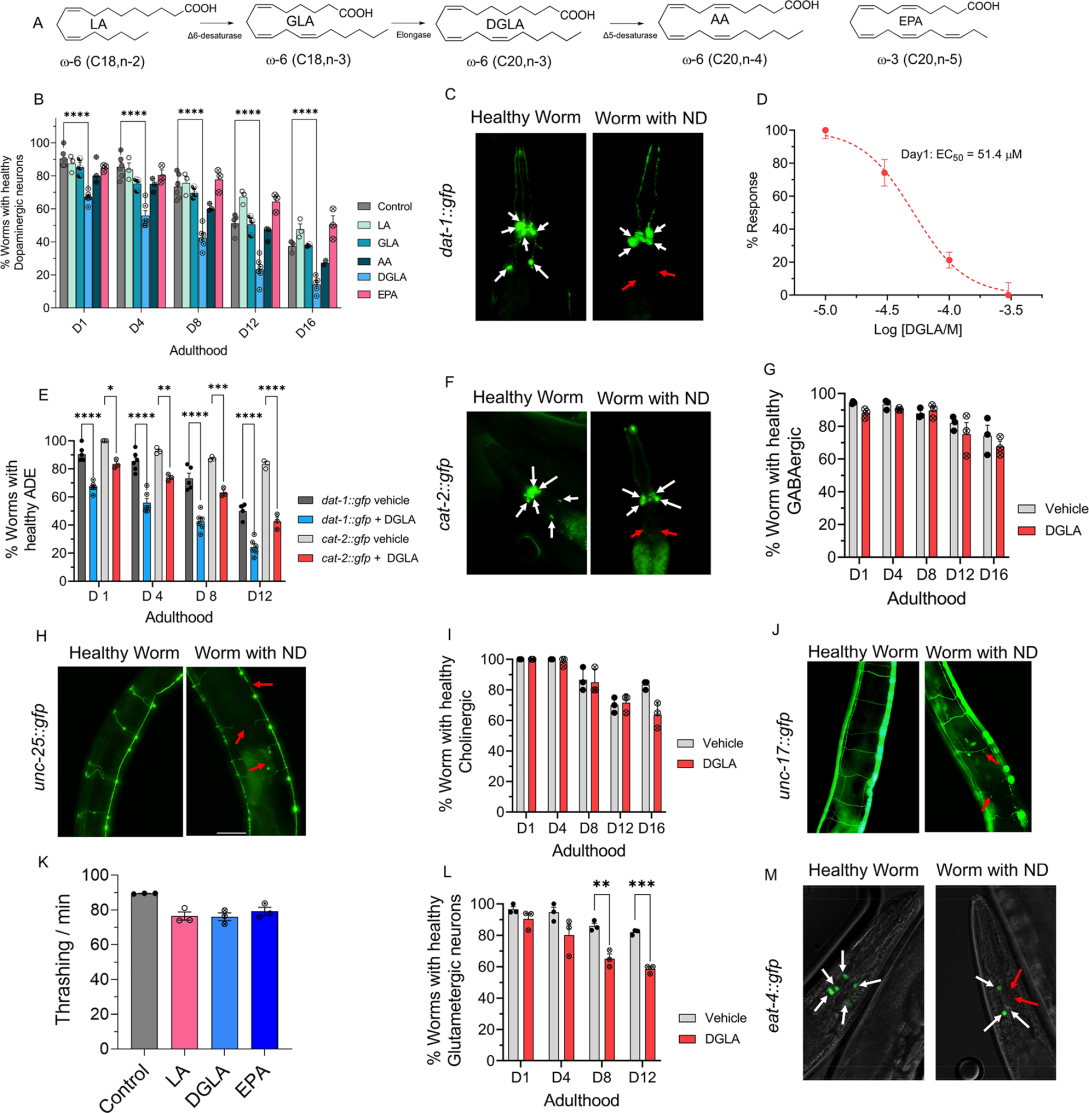
DGLA, but not other ω-3 and ω-6 PUFAs, induces degeneration, specifically in dopaminergic neurons. (A) Structure of different ω-6 and ω-3 PUFAs examined in this study. (B) Percentage (%) of worms with healthy dopaminergic neurons for *Pdat-1::gfp* with and without supplementation with 100 μM of different ω-6 and ω-3 PUFAs. (C) Fluorescent images of *Pdat-1::gfp* worms with healthy and degenerated dopaminergic neurons (white arrows represent healthy neurons, and red arrows show degenerated/disappeared neurons). (D) Dose response curve: the effect of different DGLA concentrations on degeneration of ADE neurons on day 1 adulthood. (E) Comparison of the ADE neuron degeneration in *Pdat-1::gfp* and *Pcat-2::gfp* supplemented with 100 μM DGLA. (F) Fluorescent images of *Pcat-2::gfp* worms with healthy and degenerated dopaminergic neurons (white arrows represent healthy neurons, and red arrows show degeneration/disappearance of neurons). (G) Percentage (%) of worms with healthy GABAergic neurons for *Punc-25::gfp* with and without supplementation with 100 μM DGLA. (H) Fluorescence images of *Punc-25::gfp* worm with healthy and degenerated GABAergic neurons (red arrows show different signs of neurodegeneration including ventral cord break, commissure break, and branches). (I) Percentage (%) of worms with healthy cholinergic neurons for *Punc-17::gfp* with and without supplementation with 100 μM DGLA. (J) Fluorescence images of *Punc-17::gfp* worms with healthy and degenerated cholinergic neurons (red arrows show different signs of neurodegeneration including ventral cord break, commissure break, and branches). (K) Thrashing on day 8 adulthood of wild-type raised on 100 μM LA, DGLA, and EPA. (L) Percentage (%) of worms with healthy glutamatergic neurons with *Peat-4::gfp* with and without supplementation with 100 μM DGLA. (M) Fluorescent images of *Peat-4::gfp* worms with healthy and degenerated glutamatergic neurons (white arrows represent healthy neurons, and red arrows show degenerated/disappeared neurons). All supplementations were done at the L4 stage. For all experiments, *N* = 3, and about 20 worms were tested on each trial. Two-way analysis of variance (ANOVA) and Tukey’s multiple comparison test for panels B and D; *t* test for K: *P ≤ 0.05, **P ≤ 0.01, ***P ≤ 0.001, ****P < 0.0001, nonsignificant is not shown.

Our prior lipidomic analysis showed that *C. elegans* absorbs exogenous PUFAs.^43,44^ To study the effect of dietary PUFAs on neuronal health span, we supplemented *Pdat-1::gfp* worms, in which the dopaminergic neurons are labeled by green fluorescent protein (GFP), with different ω-6 PUFAs and eicosapentaenoic acid (20:5n-3, EPA), the most abundant ω-3 PUFA in *C. elegans*,^45^ and tracked the dopaminergic neurons throughout the worm lifespan using fluorescent imaging (Figure 1A–C). Supplementation was done at the larvae stage 4 (L4) when *C. elegans* has a fully developed neuronal system, thus enabling the investigation of neurodegeneration independent of neurodevelopment.^46^ Among the tested PUFAs, only DGLA induced significant degeneration in dopaminergic neurons (Figure 1B). Furthermore, DGLA triggered degeneration in dopaminergic neurons in a dose-dependent manner with an EC_50_ = 51.4 and 31.2 μM at day 1 and day 8 adulthood, respectively (Figure 1D and Figure S1). We also showed that the vehicle control, ethanol, did not change dopaminergic neurons’ health span as compared to the control (Figure S2). In addition, we found that different types of dopaminergic neurons in the hermaphrodite had varying sensitivities to treatment with DGLA, with the ADE neurons (ADE ≫ CEP > PDE) being the most impacted (Figure S3). Moreover, loss of the GFP signal did not appear to result from transcriptional repression of the *Pdat-1::GFP* transgene induced by treatment with DGLA, since a similar trend was observed with the *Pcat-2::GFP* transgenic line upon treatment with DGLA (Figure 1E,F). We then examined whether DGLA can induce degeneration in other major types of neurons that play key roles in neurodegenerative diseases, including GABAergic, glutaminergic, and cholinergic neurons. Significant neurodegeneration was not observed in GABAergic (*Punc-25::gfp*) and cholinergic neurons (*Punc-17::gfp*) of worms supplemented with DGLA (Figure 1G–J).

These findings were further confirmed with a lack of significant changes in thrashing assays in *C. elegans* treated with DGLA (Figure 1K), which requires cholinergic and GABAergic neuron activity.^47,48^ In the case of glutamatergic neurons (*Peat-4::gfp*), treatment with DGLA caused cell loss in glutamatergic neurons only in a later stage in the *C. elegans* lifespan compared to the dopaminergic neurons (Figure 1L,M). Altogether, our results suggest that the effect of PUFAs on neurodegeneration is structurally specific.

In addition, while previous studies reported that increased lipid peroxidation could induce neurodegeneration,^49−53^ our data show that the treatment with the more peroxidizable arachidonic acid and EPA do not trigger neurodegeneration. Furthermore, our results indicate that the effect of DGLA on neurodegeneration is neuron-type selective, warranting future studies that may shine light on the molecular mechanism(s).

The remaining studies focused on the degeneration of dopaminergic neurons, as it was found that they are most sensitive to DGLA treatment. Because more robust data were obtained with transgenic *C. elegans Pdat1::gfp*, the rest of our studies were conducted using this strain. Most of the experiments were performed using day 1 and day 8 adults, enabling the determination of acute and chronic effects of DGLA treatment on neurodegeneration. Day 8 worms resemble a middle-aged population of *C. elegans*; thus, the effect of DGLA treatment on age-associated neurodegeneration can also be investigated without a significant loss (death) of the tested population, facilitating the throughput of our studies.

### DGLA Induces Neurodegeneration in Dopaminergic Neurons through Ferroptosis

**Figure 2:**
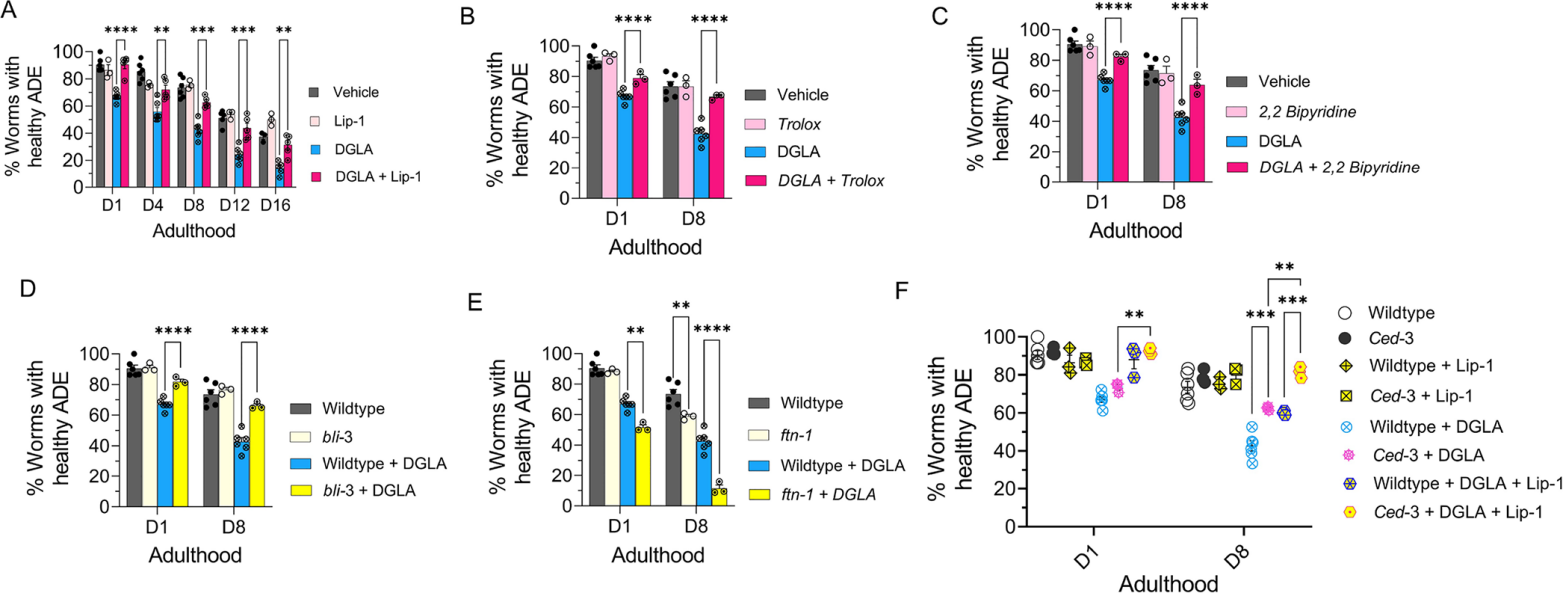
DGLA induces neurodegeneration in dopaminergic neurons via ferroptosis. (A) Percentage (%) of worms with healthy ADE neurons of worms exposed to 100 μM DGLA ± 250 μM liproxstatin-1. (B) Percentage (%) of worms with healthy ADE neurons in wild-type *C. elegans* treated with 100 μM DGLA ± 500 μM Trolox (vitamin E). (C) Percentage (%) of worms with healthy ADE neurons for *Pdat-1:*:gfp worms treated with 100 μM DGLA ± 100 μM 2,2′-bipyridine. (D) Percentage (%) of worms with healthy ADE neurons in *Pdat-1::gfp* and *Pdat-1::gfp;bli-3* worms treated with 100 μM DGLA. (E) Percentage (%) of worms with healthy ADE neurons for *Pdat-1::gfp* and *Pdat-1::gfp;ftn-1* worms treated with 100 μM DGLA. (F) Percentage (%) of worms with healthy ADE neurons with *Pdat-1::gfp* and *Pdat-1::gfp;ced-3* worms treated with 100 μM DGLA ± 250 μM liproxstatin-1. All supplementations were done at the L4 stage. Two-way analysis of variance (ANOVA), Tukey’s multiple comparison test. *P ≤ 0.05, **P ≤ 0.01, ***P ≤ 0.001, ****P < 0.0001; NS, not significant. DGLA, Dihomo-γ-linolenic acid; LA, linoleic acid; EPA, eicosapentaenoic acid; Lip-1, liproxstatin-1.

Recent studies show that treatment with DGLA can induce ferroptosis in germ cells and cause sterility in *C. elegans*.^17^ To test whether treatment with DGLA induces degeneration in dopaminergic neurons through ferroptosis, *Pdat-1::gfp* expressing worms were cotreated with DGLA and liproxstatin-1 (Lip-1), a radical-trapping antioxidant and ferroptosis inhibitor.^54^ While C. *elegans* treated with Lip-1 alone showed no significant effect on age-associated degeneration of dopaminergic neurons as compared to the vehicle control, cotreatment of DGLA with Lip-1 fully rescued the neurodegeneration triggered by DGLA in day 1 adults and largely rescued DGLA-induced neurodegeneration in day 8 adults (Figure 2A). Encouraged by these results, we examined neurodegeneration caused by ferroptosis in DGLA-treated worms using pharmacological and genetic approaches. An increase in the labile iron(II) pool and membrane lipid peroxidation are molecular hallmarks of ferroptosis.^6,55^ Therefore, we tested whether treatment with Trolox, a water-soluble form of vitamin E and lipid peroxidation inhibitor, and 2,2′-bipyridine, an iron(II) chelator, alleviates DGLA-induced neurodegeneration. Cotreatment with either Trolox or 2,2′-bipyridine rescued DGLA-induced neurodegeneration, suggesting that both the labile iron(II) pool and membrane lipid peroxidation are involved in DGLA-induced neurodegeneration (Figure 2B,C). To specifically investigate whether ferroptosis is involved in DGLA-induced neurodegeneration, a genetic approach was also pursued.

Previous studies have shown that the nicotinamide adenine dinucleotide phosphate (NADPH) oxidase (NOX) family of superoxide-producing enzymes (NOX/DUOX) plays a critical role in ferroptosis in mammals and can exacerbate dopaminergic neurotoxicity triggered by ferroptosis inducers.^4,56^ In addition, ferritin (FTN) is also a key ferroptosis regulatory protein, and the genetic knockout of FTN has been shown to sensitize *C. elegans* to ferroptosis.^17,57^ To further test whether ferroptosis is involved in DGLA-induced neurodegeneration, two new transgenic *C. elegans* strains were created by crossing the *Pdat-1::gfp* with transgenic strains that carry either a loss of function of *bli-3* (*C. elegans* homologue of NOX) mutant or genetic knockout of *ftn-1* (Figure 2D,E and Figure S4). Our results indicated that the loss of function of the *bli-3* mutant reduced the degeneration of dopaminergic neurons triggered by DGLA (Figure 2D). Worms with loss of function mutations of BLI-3 attenuated the ability to generate reactive oxygen species, thus minimizing lipid peroxidation and, as a result, reduced ferroptosis.^17,58^ This result further confirms the pharmacological observation after supplementing worms with the lipophilic antioxidant vitamin E (Trolox), which led to the suppression of neurodegeneration in DGLA-treated worms (Figure 2B). Furthermore, genetic knockout of *ftn-1* enhanced DGLA-induced neurodegeneration, suggesting that DGLA requires the labile iron(II) pool to exert its effect on dopaminergic neurons (Figure 2E). Our data strongly suggest that DGLA causes the degeneration of dopaminergic neurons at least partly through ferroptosis. As illustrated in Figure 2A, while Lip-1 fully rescued DGLA-induced neurodegeneration for day 1 adults, such rescuing effect diminished as *C. elegans* aged. Furthermore, the EC_50_ of DGLA in triggering neurodegeneration for day 1 and day 8 adults is different. Therefore, we hypothesize that chronic treatment with DGLA induces other programmed cell-death pathways, like apoptosis, leading to neurodegeneration.

To test whether DGLA also induces neurodegeneration through apoptosis,^59^ an additional transgenic strain was developed. The CED-3 protein, a key enzyme involved in apoptosis, was genetically knocked out in worms in which the dopaminergic neurons were labeled by GFP^17,60^ to create a transgenic line, *Pdat-1::gfp;ced-3(n717)*. Interestingly, while no significant difference was observed between *Pdat-1::gfp;ced-3(n717)* and *Pdat-1::gfp* worms treated with DGLA at day 1 adulthood, worms that had the *ced-3* genetic knockout demonstrated partial rescue from dopaminergic neuron degeneration induced by DGLA (Figure 2F) at day 8 of adulthood. Furthermore, the *ced-3* knockout worms at day 8 adulthood that were cotreated with Lip-1 were fully rescued from dopaminergic neurodegeneration induced by DGLA (Figure 2F). These results could explain the differences observed for day 1 and day 8 worms treated with Lip-1 and DGLA (Figure 2A), as well as the differences in the EC_50_ of DGLA-induced neurodegeneration between day 1 adults (EC_50_ = 51.4 μM) and day 8 adults (EC_50_ = 31.2 μM) (Figure 1D). The lower EC_50_ for DGLA-induced neurodegeneration for day 8 adults suggests that other neurodegenerative mechanisms (i.e., apoptosis and autophagy) are involved and could either synergize or provide an additive effect with DGLA-induced neurodegeneration. Together, these results suggest that dietary DGLA induces neurodegeneration via ferroptosis in early adulthood, and both ferroptosis and additional mechanism(s), such as apoptosis, are induced by DGLA in dopaminergic neurodegeneration in middle-aged *C. elegans*.

### Downstream Metabolites of DGLA Are Key Players in Neurodegeneration Induced by DGLA Treatment

**Figure 3:**
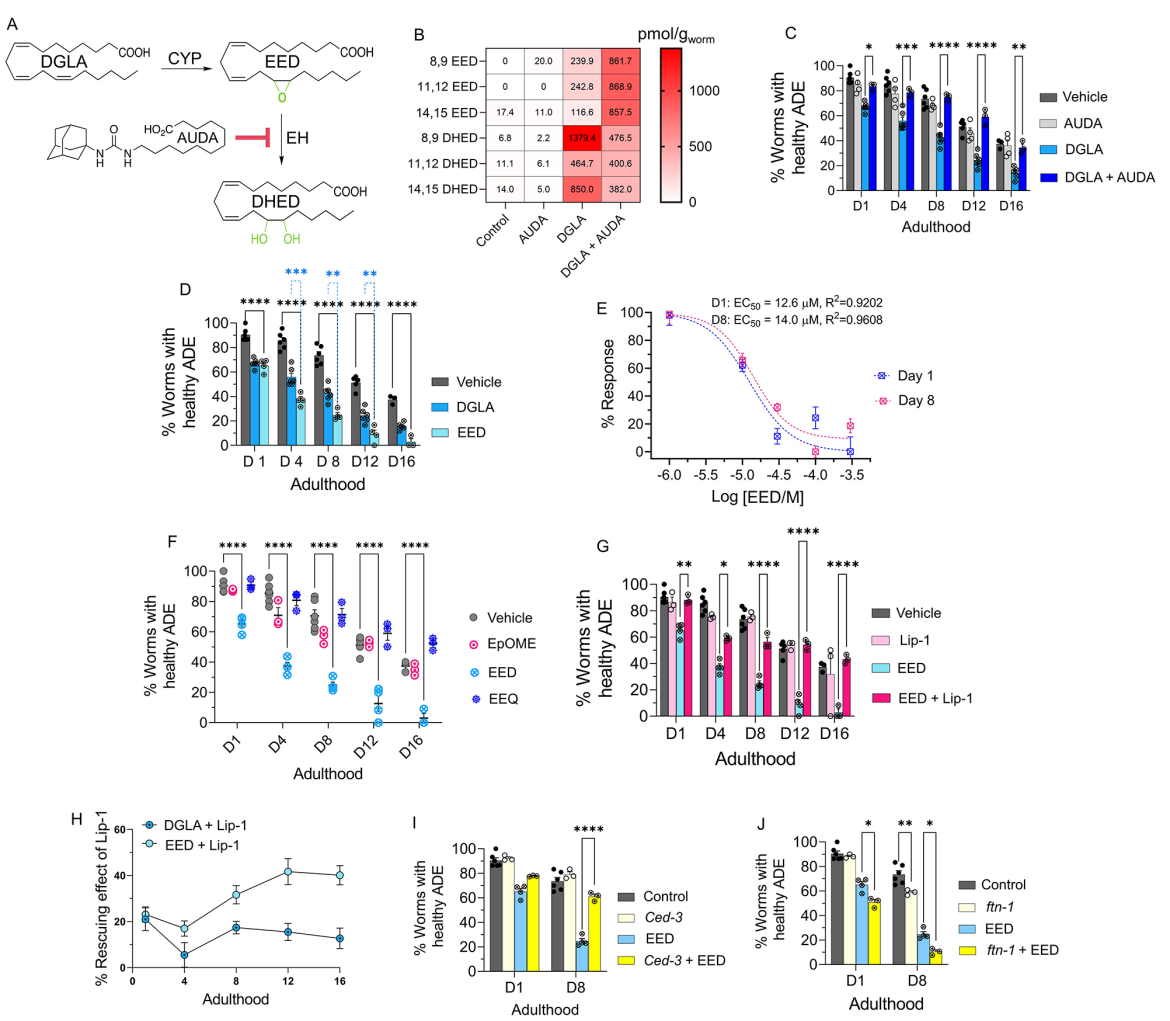
EED, epoxy metabolites downstream of DGLA, induce neurodegeneration by ferroptosis. (A) DGLA is metabolized to EED and DHED through the CYP and epoxide hydrolase enzymes, respectively, and AUDA inhibits epoxide hydrolase. (B) Oxylipin profile representing the pmol/g of EED and DHED regioisomers in worms treated with 100 μM DGLA ± 100 μM AUDA compared to control. (C) Percentage (%) of worms with healthy ADE neurons for *Pdat-1::gfp* treated with 100 μM DGLA ± 100 μM AUDA. (D) Percentage (%) of worms with healthy ADE neurons for *Pdat-1::gfp* worms treated with 100 μM DGLA and 100 μM EED. (E) Dose response curve: effect of different concentrations of EED on degeneration of ADE neurons on day 1 and day 8 adulthood. (F) Percentage (%) of worms with healthy ADE neurons in *Pdat-1::gfp* worms treated with 100 μM of different Ep-PUFAs, EpOME, and EEQ. (G) Percentage (%) of worms with healthy ADE neurons of worms treated with 100 μM DGLA ± 100 μM liproxstatin-1. (H) Comparison of the effect of 250 μM liproxstatin-1 on *Pdat-1::gfp* worms treated with 100 μM DGLA compared to 100 μM EED. (I) Percentage (%) of worms with healthy ADE neurons with *Pdat-1::gfp* and *Pdat-1::gfp;ced-3* worms treated with 100 μM. (J) Percentage (%) of worms with healthy ADE neurons for *Pdat-1::gfp* and *Pdat-1::gfp;ftn-1* worms treated with 100 μM DGLA; all supplementations were done at the L4 stage. Two-way analysis of variance (ANOVA), Tukey’s multiple comparison test. *P ≤ 0.05, **P ≤ 0.01, ***P ≤ 0.001, ****P < 0.0001; without *, not significant. DGLA, Dihomo-γ-linolenic acid; EED, epoxyeicosadienoic acids; DHED, dihydroxyeicosadienoic acids; CYP, cytochrome P450; EH, epoxide hydrolase; AUDA, 12-(1-adamantane-1-yl-ureido-) dodecanoic acid; Lip-1, liproxstatin-1; EpOME, epoxyoctadecenoic acids; EEQ, epoxyeicosatetraenoic acid.

In mammals, DGLA and other PUFAs are monooxygenated by cytochrome P450 enzymes (CYPs) to hydroxy- and epoxy-PUFAs. Epoxy-PUFAs are further hydrolyzed by epoxide hydrolases (EHs) to the dihydroxy-PUFAs (Figure 3A).^18^ Numerous animal and human studies have demonstrated that endogenous levels of CYP and EH metabolites produced from various PUFAs are highly correlated to the dietary intake of the corresponding PUFAs,^61−63^ in stark contrast to metabolites generated by cyclooxygenases and lipoxygenases, which are less correlated.^64−66^ Both epoxy- and dihydroxy-PUFAs are key signaling molecules for mammalian physiology, including, but not limited to, neuroprotection.^18,67,68^ Therefore, we hypothesized that DGLA primarily induces ferroptosis-mediated neurodegeneration via its CYP-EH metabolites, a previously unexplored area. To test this hypothesis, we first investigated whether the CYP-EH metabolism is involved in DGLA-induced ferroptosis-mediated neurodegeneration by investigating how treatment with DGLA impacts CYP-EH metabolism. Our results indicated that treatment with 100 μM DGLA increased the whole animal endogenous levels of the corresponding epoxyeicosadienoic acid (EED) and dihydroxyeicosadienoic acid (DHED) to ∼200 and ∼800 pmol/g, respectively (regioisomer-dependent Figure S5), using our oxylipin analysis (Pourmand et al., unpublished, see Experimental Methods in the SI). These results were similar to the endogenous levels of EPA CYP-EH metabolites, epoxyeicosatetraenoic acid (EpETEs) and dihydroxyeicosatetraenoic acid (DHETE) which are 50–919 and 0–458 pmol/g, respectively (regioisomer-dependent), in intact *C. elegans*, suggesting that the increased level of EED and DHED is physiologically relevant (Figure 3B and Figure S5). Therefore, we sought to determine whether these downstream metabolites (EED and DHED) are key mediators for neurodegeneration induced by treatment with DGLA. Transgenic *C. elegans* (*Pdat-1::gfp*) were cotreated with DGLA and 12-(1-adamantane-1-yl-ureido-)dodecanoic acid (AUDA, 100 μM), an EH inhibitor with selective action to inhibit the function of CEEH1 and CEEH2 (*C. elegans* EH1 and EH2 isoforms).^69^ AUDA treatment increased the level of EED and decreased the DHED *in vivo* concentration and fully rescued dopaminergic neurodegeneration induced by DGLA, suggesting that the CYP/EH-derived downstream metabolites of DGLA play a critical role in DGLA-induced neurodegeneration (Figure 3B,C).

Nonetheless, these results do not discriminate between AUDA’s ability to stabilize the level of EED *in vivo* for the observed rescue or to block the production of DHED metabolite that result from inhibiting *C. elegans* EHs (Figure 3B and Figure S6). To distinguish between the latter two possibilities, we synthesized both EED and DHED and tested their effects in *C. elegans* following the procedures in previous reports.^70−73^ Treatment with 100 μM EED at the L4 stage induced a more severe neurodegenerative phenotype than treatment with DGLA at the same concentration in the dopaminergic neurons in all tested ages (Figure 3D), with a much lower EC_50_ (12.6 vs 51.4 μM) as compared to DGLA on day 1 adult (Figures 3E and 1D). The same trend was observed in glutamatergic neurons when comparing treatments of EED and DGLA, and similar to DGLA treatment, no significant neurodegeneration was observed in GABAergic and cholinergic neurons after treatment with EED (Figure S7).

To test whether the effect of EED is structurally specific, C18:1 epoxyoctadecenoic acid (EpOME), an epoxy metabolite of LA, and a more peroxidizable C20:4 epoxyeicosatetraenoic acid (EEQ), an epoxy metabolite of EPA, were examined (Figure 3F) and had no effects on neurodegeneration. These results indicate that the effect of epoxy-PUFAs on neurodegeneration is specific to EED, but not other epoxy-PUFAs. Similar to the neurodegeneration induced by DGLA, cotreatment with Lip-1 rescued neurodegeneration caused by EED, and Lip-1 was more effective in alleviating EED-induced neurodegeneration compared to DGLA-induced neurodegeneration (Figure 3G,H). Furthermore, like DGLA, neuronal degeneration induced by EED was not rescued by a genetic knockout of *ced-3.* Genetic knockout of *ftn-1* escalates the effect of EED in both day 1 and day 8 adults, again suggesting that ferroptosis plays a critical role in EED-induced neurodegeneration (Figure 3I,J).

**Figure 4:**
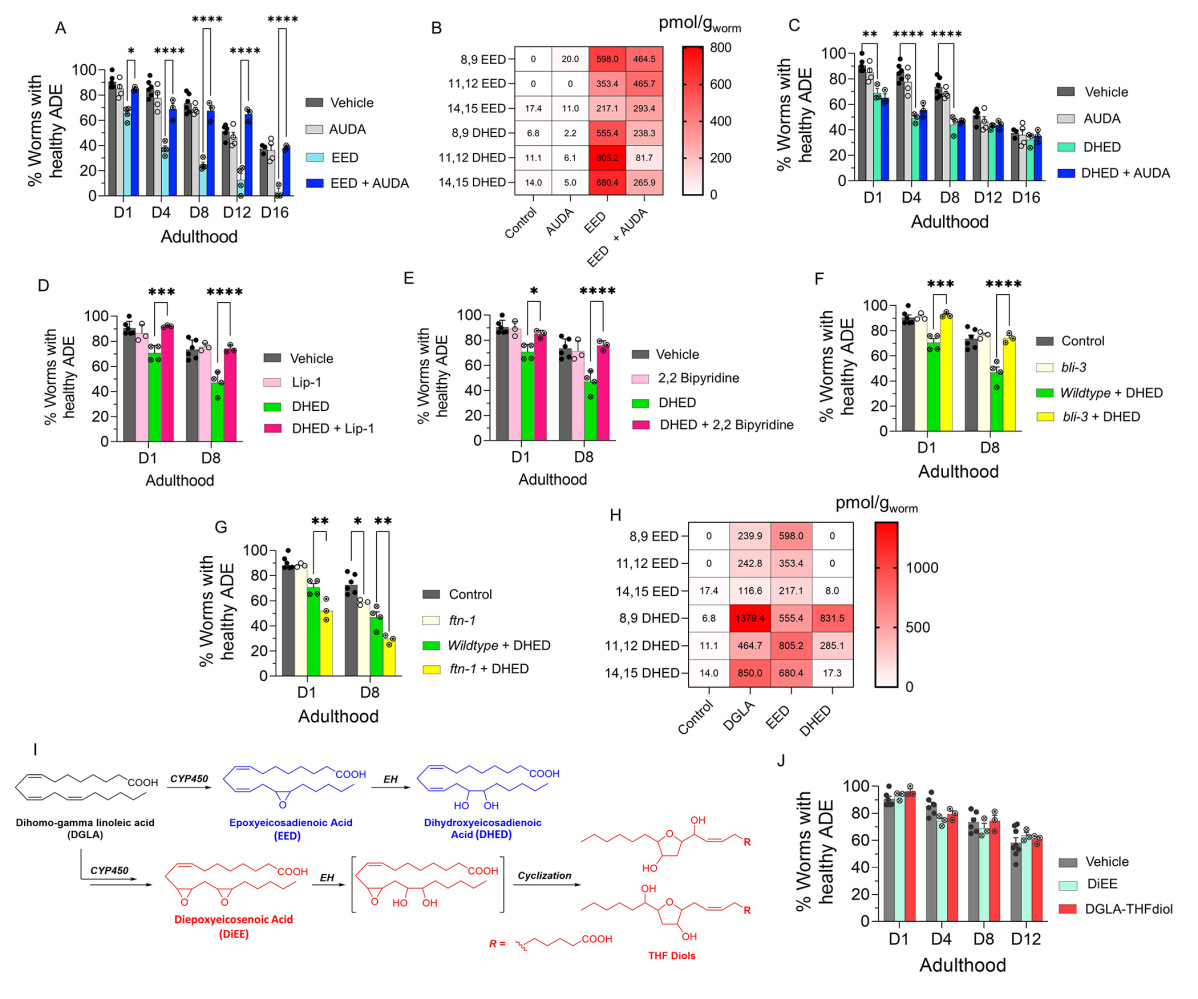
DHED, dihydroxy fatty acid downstream of DGLA/EED, is key candidates for neurodegeneration induced by DGLA in dopaminergic neurons. (A) Percentage (%) of worms with healthy ADE neurons in *Pdat-1::gfp* worms treated with 100 μM EED ± 100 μM AUDA. (B) Oxylipin profile representing pmol/g of EED and DHED regioisomers in worms treated with 100 μM EED ± 100 μM AUDA compared to control. (C) Percentage (%) of worms with healthy ADE neurons for *Pdat-1::gfp* treated with 100 μM DHED ± 100 μM AUDA. (D) Percentage (%) of worms with healthy ADE neurons of worms exposed to 100 μM DHED ± 250 μM liproxstatin-1. (E) Percentage (%) of worms with healthy ADE neurons for *Pdat-1:*:gfp worms treated with 100 μM DHED ± 100 μM 2,2′-bipyridine. (F) Percentage (%) of worms with healthy ADE neurons for *Pdat-1::gfp* and *Pdat-1::gfp;ftn-1* worms treated with 100 μM DHED. (G) Percentage (%) of worms with healthy ADE neurons in *Pdat-1::gfp* and *Pdat-1::gfp;bli-3* worms treated with 100 μM DHED. (H) Oxylipin profile representing the pmol/g of EED and DHED regioisomers in worms treated with 100 μM DGLA, EED, and DHED compared to control. (I) Two possible metabolisms of DGLA through the CYP/EH pathways; the alternative metabolism is that CYP can do two consecutive oxidations (or under oxidative stress) to yield diepoxies EED, after which EH will open one epoxide which under physiological conditions can cyclize to THF diols. (J) Percentage (%) of worms with healthy ADE neurons for *Pdat-1::gfp* treated with 100 μM DiEE and 100 μM DGLA-THF diol. All supplementations were done at the L4 stage. Two-way analysis of variance (ANOVA), Tukey’s multiple comparison test. *P ≤ 0.05, **P ≤ 0.01, ***P ≤ 0.001, ****P < 0.0001; without *, not significant. DGLA, Dihomo-γ-linolenic acid; EED, epoxyeicosadienoic acid; DHED, dihydroxyeicosadienoic acid; CYP, cytochrome P450; EH, epoxide hydrolase; AUDA, 12-(1-adamantane-1-yl-ureido-) dodecanoic acid; DiEE, diepoxyeicosadienoic acid.

Our results further suggested that DGLA metabolites are lipid mediators responsible for the effect of DGLA on neurodegeneration. Inhibition of EED hydrolysis using an EH inhibitor (AUDA) resulted in the rescue of EED-induced neurodegeneration in *C. elegans* (Figure 4A). The oxylipin profile of worms cotreated with EED and AUDA at 100 μM shows that blocking the metabolism of epoxy-PUFAs to dihydroxy-PUFAs, specifically EED to DHED with AUDA, stabilizes endogenous levels of epoxy-PUFAs including EED and decreases the *in vivo* levels of dihydroxy-PUFAs and DHED (Figure 4B and Figure S8). Altogether, our data further suggest that specific DGLA downstream metabolites, either EED or DHED, are responsible for DGLA-mediated neurodegeneration.

We further corroborated our hypothesis by supplementing *Pdat-1::gfp* worms with 100 μM DHED, which showed significant neurodegeneration compared to the vehicle control (Figure 4C). Intriguingly, cotreatment with AUDA and DHED did not alleviate neurodegeneration induced by DHED, further confirming that DHED is likely the main driver of dopaminergic neurodegeneration in our model (Figure 4C). Cotreatment with AUDA alleviated DGLA-induced neurodegeneration likely by blocking the formation of DHED. In addition, cotreatment with Lip-1 and 2,2-bipyridine rescued the neurodegeneration caused by DHED (Figure 4 D,E). Furthermore, the loss of function of the *bli-3* mutant also reduced the degeneration of dopaminergic neurons triggered by DHED, and genetic knockout of *ftn-1* augments DHED-induced neurodegeneration (Figure 4F,G). Together, these results suggest that the labile iron(II) pool and subsequent ferroptosis are involved in the effect of DHED on dopaminergic neurons. It is noteworthy that we did not observe more severe neurodegeneration induced by DHED as compared to EED supplementation. This effect may be due to the difference in lipid transport mechanism between dihydroxy-PUFAs, PUFAs, and Ep-PUFAs, as suggested by a previous study.^74^ DHED is not absorbed as well as EED and thus is not as potent at the same concentration. This hypothesis was confirmed by oxylipin profiling, which showed significantly lower DHED levels (especially for 11,12 and 14,15 DHED) in worms treated with 100 μM DHED as compared to those treated with 100 μM EED or 100 μM DGLA (Figure 4D and Figure S9). While DGLA and EED exhibit a continuous increase in dopaminergic neurodegeneration over their lifespan, DHED exhibited a plateau after day 8, which suggests the presence of a possible mechanism for removal of the offending agent, either inducing downstream metabolism or activating lipid transport of DHED after chronic treatment. Our data strongly suggest that it induces neurodegeneration through its downstream metabolites. Beyond DHED, there have been a few reports of other downstream CYP-mediated metabolites, namely, epoxy-hydroxy-PUFA and diepoxy-PUFAs, which ultimately undergo spontaneous intramolecular cyclization in physiological conditions to form an understudied class of metabolites, the tetrahydrofuran-diols (THF-diols). These THF-diols could be a new class of lipid mediators in mammals.^75,76^ To examine these potential mediators of biological activity, isomeric vicinal diepoxyeicosenoic acid (DiEE) and its corresponding THF-diol (DGLA-THF-diol) were synthesized and incubated with the worms (see the Experimental Section in the SI and Figure 4E). However, no significant loss was observed in dopaminergic neurons in worms treated with 100 μM DiEE and its corresponding THF-diol compared with the vehicle control (Figure 4F). These results strongly suggest that DHED constitutes a novel class of lipid mediators that induce neurodegeneration largely mediated by ferroptosis. In addition, EpETE and EpOME produced from EPA and LA, respectively, showed no effect on neurodegeneration, which further corroborates that the effect of DHED on ferroptosis-mediated neurodegeneration is structurally selective. Furthermore, the results obtained from the treatment with more peroxidizable EPA and EpETE indicate that the effect of DHED is not due to an increase in the level of peroxidation of the cell membrane, a known mechanism that sensitizes ferroptosis.^15,77−79^ As a whole, the results summarized above are in contrast with reports on the effect of PUFAs^80−82^ or their metabolites, such as lipoxygenase’s metabolites,^54,77,81^ ether lipids,^83^ etc., on ferroptosis.

## Discussion

Our studies revealed that DHED, the CYP-EH metabolite of DGLA, is a novel class of lipid molecules that trigger ferroptosis-mediated degeneration in select neuron types in *C. elegans*. Our study addresses critical gaps in knowledge in the field of lipid pharmacology, neurodegeneration, and ferroptosis, including how ω-6 PUFAs may trigger neurodegeneration and the identity of endogenous signaling molecules that induces ferroptosis-mediated neuronal cell death. Most research investigates the beneficial effects of ω-3 PUFA supplementation in neurodegenerative diseases, with contradictory findings.^26,27,30^ Few studies have tested the effect of ω-6 PUFAs on neurodegeneration.^28^ This is of high interest since ω-6 PUFA levels are typically high in western diets. Our findings in *C. elegans* demonstrate that, unlike other PUFAs, the ω-6 DGLA induces ferroptosis-mediated degeneration specifically in dopaminergic and to a lesser extent in glutaminergic neurons. Recent reports suggest that PUFAs play a critical role in ferroptosis; treating cells with PUFAs, their ether-lipid metabolites, and lipoxygenase metabolites, hydroperoxyeicotetraenoic acids, sensitizes cells to ferroptosis, but they do not induce ferroptosis themselves.^54,83^ Although synthetic compounds such as erastin, RSL-3, and natural products such as α-eleostearic acid have been identified as agents that can induce ferroptosis, the specific endogenous mediators that regulate the upstream pathway of ferroptosis remain unknown.^13−15,31^ Our results indicate that DGLA induces ferroptosis-mediated neuronal death likely through its downstream endogenous CYP-EH metabolites, DHED, and EH plays a critical role in modulating DGLA-mediated ferroptosis. Our study complements the previous elegant work showing that DGLA induces ferroptosis in germline and cancer cells.^17^ The identification of potential lipid signaling molecules represents a critical first step in investigating the molecular mechanism behind the effects of PUFAs on ferroptosis-mediated neurodegeneration.

Recent reports demonstrated that the expression of soluble EH, a human orthologue of CEEH 1/2, is upregulated in patients with neurodegenerative diseases including Parkinson’s disease and Alzheimer’s disease, and inhibition of soluble EH is beneficial for neurodegeneration in multiple neurodegenerative diseases animal models.^18,84−87^ While the specific role of soluble EH in neurodegeneration is largely unknown, these studies suggested that the epoxy-fatty acids, the substrates of soluble EH, are neuroprotective, and the corresponding downstream EH metabolites dihydroxy-fatty acids have no effect, although a few studies in cell and animal models have shown that these dihydroxy-fatty acids can have detrimental or toxic effects on cells.^88,89^ Our results provide an alternate perspective of how neurodegeneration could be regulated endogenously by modulating EH activity to increase ferroptotic metabolites, namely, DHED, which has seldom been studied.

Our finding that DHED modulate ferroptosis-mediated neurodegeneration challenges the current paradigm in the field. Numerous studies demonstrated that membrane lipid composition and lipid peroxidation are essential for ferroptosis.^4,77,90,91^ Supplementation with PUFAs and their metabolites, particularly those metabolites with a higher degree of unsaturation, sensitizes cells to ferroptosis.^4^ However, unlike synthetic compounds such as erastin, RSL-3, etc., these lipid molecules do not trigger ferroptosis but rather act downstream of ferroptosis pathways by increasing the rate of membrane lipid peroxidation.^4,92,93^ This phenomenon is further supported by studies showing that supplementation with a monosaturated fatty acid, such as oleic acid, desensitizes cells from ferroptosis.^78^ In contrast, our results show that a specific ω-6 DGLA metabolite, DHED, induces ferroptosis, while other PUFAs (AA and EPA) and EPA metabolites (EEQ), with a higher degree of unsaturation, do not trigger ferroptosis. This observation aligns with a recent study showing that although EPA and AA supplementation are more deleterious in peroxide-induced whole-body oxidative stress, they cannot trigger ferroptotic germline cell death in *C. elegans*.^43^

Our data using Lip-1 supplementation, along with the use of transgenic strains carrying a loss of function *ftn-1* mutation, suggest that DHED could trigger lipid peroxidation in the ferroptosis pathway. However, it is unlikely that DHED induces ferroptosis-mediated neurodegeneration by undergoing peroxidation itself, as discussed above, because supplementation with AA, EPA, and EEQ, which are more prone to lipid peroxidation, has minimal or no effects in our neurodegenerative assays. In addition, it has been reported that dihydroxy-PUFAs are unable to incorporate into cell membranes,^94^ which suggests that DHED has a distinct mechanism for modulating ferroptosis compared to other PUFAs. This is because PUFAs with high degrees of unsaturation can propagate membrane lipid peroxidation during ferroptosis upon incorporation into the cell membrane. Although the exact mechanism underlying DHED induction of ferroptosis-mediated neurodegeneration is largely unknown, and falls beyond the scope of this study, we propose that DHED may interact with potential receptor proteins to activate the upstream ferroptosis pathway, leading to iron-mediated lipid peroxidation. This corroborates our finding from the experiments with Lip-1 and transgenic loss of function *ftn-1* strains, which indicate a critical role for lipid peroxidation in DHED-induced neurodegeneration. While DHED has not been extensively studied, 9,10-dihydroxyoctadecenoic acid (DiHOME) and 12,13-DiHOME, which are dihydroxy-metabolites of LA, activate peroxisome proliferator-activated receptor (PPAR) gamma and transient receptor potential vanilloid 1 (TRPV1), respectively.^95,96^ In addition, 14,15-dihydroxyeicosatrienoic acid, a dihydroxy-metabolite of AA, also activates PPAR alpha.^97^ All of these proteins have been associated with ferroptosis.^98−101^ Therefore, DHED could modulate ferroptosis-mediated neurodegeneration by interacting with one of these proteins or similar proteins. Alternatively, although DHED is less likely to be incorporated into the cell membrane, it could still be localized into specific subcellular compartments such as mitochondria, the endoplasmic reticulum (that contains the largest pool of lipids in cells), and lysosomes, where DHED could be peroxidized and propagate lipid peroxidation, leading to ferroptosis.^13,54,102−105^ Currently, our laboratory is conducting a variety of genetic experiments to identify potential receptor proteins for DHED and synthesizing deuterated DHED to investigate whether DHED peroxidation is necessary for their action in ferroptosis-mediated neurodegeneration.

In this study, we employed an approach that comprised a simple animal model, an inhibitor of a metabolic enzyme, synthesized lipid metabolites, and targeted metabolomics to systematically investigate the crosstalk between lipid metabolism, neurodegeneration, and ferroptosis in a highly efficient way. We have not only identified the key mediator for ferroptosis-mediated neurodegeneration but have also revealed that DGLA and its metabolites have more pronounced effects on dopaminergic neurons, mild effects on glutaminergic neurons, and no effects on cholinergic and GABAergic neurons in *C. elegans*. Our results complement previous studies by Zille et al., which showed that different cell types could have distinct regulatory pathways for ferroptosis.^106^ While the specific mechanism behind why DGLA and its metabolites, DHED, are more detrimental to dopaminergic neurons remains unknown, Fonseca et al. reported a similar vulnerability of different neuron types in response to biomechanical injury and suggested that such observation could be due to different physiological regulatory mechanisms between different neuron types.^107^ Such neuron-type-specific effects triggered by DGLA and DHED warrant future investigation to uncover potential new neurodegeneration mechanisms.

However, investigating ferroptosis to understand differential ferroptosis mechanisms between tissues requires studies being carried out at the system level, which is challenging owing to the lack of appropriate genetic and imaging tools. The genetic malleability of *C. elegans* provides a suitable platform for the study of ferroptosis in a tissue-specific manner. Furthermore, as illustrated in Table S1, most cell lines do not express soluble EH, a human orthologue of CEEHs, and studies have demonstrated that different tissues express CYP enzymes and soluble epoxide hydrolase differently.^35−38^ Therefore, a whole animal approach is more appropriate for us to explore this novel mechanism, and *C. elegans* provides a simple animal model. In addition, the adaptability to high-throughput studies of *C. elegans* allows us quickly to dissect complicated pathways. As such, it is possible to explore how ferroptosis can be regulated differentially by endogenous signaling molecules, such as DHED, between cell-types in an intact organism. Furthermore, the chemical tools developed and utilized for this study lead to the exploration of novel hypotheses that aim to unravel PUFAs’ effects on organismal physiology, an area that not only is understudied but also is challenging to execute in mammalian models and humans.

## Conclusion

Oxidized lipid metabolites are key mediators for organismal physiology. Ferroptosis, characterized by an increase in iron-dependent lipid peroxidation, could be a novel mechanism for neurodegeneration. In this study, we reported that exogenous DGLA triggers neurodegeneration predominantly in dopaminergic neurons via its downstream cytochrome P450-epoxide hydrolase (CYP-EH) metabolite, dihydroxyeicosadienoic acid (DHED). The observed neurodegeneration induced by DGLA/DHED is likely mediated by ferroptosis at the early stages and a combination of ferroptosis and apoptosis after chronic treatment with DGLA/DHED. This study revealed that CYP-EH polyunsaturated fatty acid (PUFA) metabolism is one of the key intrinsic regulatory mechanisms of ferroptosis-mediated neurodegeneration, and EH could be a novel target for ferroptosis-mediated diseases.

## Supporting Information Available

Experimental methods and materials, supplemental figures, and synthesis and characterization of DGLA metabolites (PDF)
Table S1: soluble EH expression in cell lines (XLS)
Transparent Peer Review report available (PDF)
