# Supplementary material for: Dihydroxy-Metabolites of Dihomo-γ-linolenic Acid Drive Ferroptosis-Mediated Neurodegeneration
Source: ACS Cent Sci. 2023 Mar 16;9(5):870–82. doi: 10.1021/acscentsci.3c00052 (PMC10214511; doi:10.1021/acscentsci.3c00052)
Supplement: Supplementary file 3 — oc3c00052_si_003.pdf [file oc3c00052_si_003.pdf]

MICHIGAN STATE UNIVERSITY

Feb 20th, 2023

Prof. Christopher Chang
Deputy Editor
ACS Central Science
Fax: (202) 354-5306
Email: chang-office@centralscience.acs.org

Dear Prof. Chang,

This letter is regarding the re-submission of the revised manuscript entitled "Dihydroxy-Metabolites of Dihomo-gamma-linolenic Acid Drive Ferroptosis-Mediated Neurodegeneration" (Manuscript ID: oc-2023-000526) to ACS Central Science. We appreciate all the positive comments regarding this work, particularly, reviewer 1 thinks this research study is suitable for media coverage or a First Reactions (a News & Views piece in the journal). We also sincerely thank the reviewers and editor for their thoughtful suggestions. We have addressed all the comments in the revised manuscript and included a point-by-point response to the comment at the end of this letter.

We believe the revised manuscript has been substantially improved after we addressed these valuable comments from the reviewers and the editor. In the resubmission, we have included the revised manuscript, the revised manuscript with the modifications highlighted, and a point-by-point response to the comments. Should you have any questions or need additional information, please do not hesitate to contact us. Thank you very much for your consideration.

Yours sincerely,

Kin Sing Stephen Lee
Assistant Professor
Department of Pharmacology and Toxicology
Department of Chemistry
Institute of Integrative Toxicology
Michigan State University
B330 Life Science Building
1355 Bogue St
East Lansing, MI-48824

Department of Pharmacology and Toxicology

Michigan State University
B330 Life Science Building
1355 Bogue St
East Lansing, MI 48824

517-884-1813
phmtox.msu.edu

MSU is an affirmative-action, equal-opportunity employer.

Response to the reviewers (The response to the comment was in bold):

Reviewer1:

The authors show that the PUFA DGLA induces cell death through ferroptosis in dopaminergic neurons in C elegans. The effect was specific to DGLA not other PUFAs, suggesting a specific metabolic fate of DGLA is involved. The neurodegeneration induced by DGLA was suppressed by the epoxide hydrolase inhibitor AUDA, suggesting that the dihydroxy product of the hydrolase is key for the neurodegeneration. It is curious the lip1 rescued the neurodegeneration induced by EED, as this implies that EED acts through driving a radical-mediated propagation of lipid peroxides, which seems chemical implausible, at least compared to DGLA itself. But the ftn1 dependence of EED seems to support this. The same holds for DHED, the hydroxy product of the epoxide hydrolysis. There is no discussion of this seeming paradox. I can imagine that the dihydroxymetabolite DHED has a distinct subcellular localization and the remaining bis-allylic carbon in DHED is subject to radical-mediated peroxidation in this specific subcellular localization, which drives the death of dopaminergic neurons. An alternative explanation is that lip-1 and ftn1 block production of a necessary driving factor, such as some lipid peroxides that synergies with DHED. But either way, it seems critical that DHED undergo further peroxidation to drive neurodegeneration and ferroptosis. The authors could test this by making di-deuterated analog of DHED where the deuterium atoms are at the bis-allylic position, which should block its ability cause neurodegeneration. But I think this is beyond the scope of the current paper, as that would be an involved process to synthesize. Nonetheless I think some discussion of this point is important, otherwise it seems paradoxical.

Response: **We appreciate the reviewer's positive feedback and constructive comments, which helped us to clarify and strengthen our arguments. We have included an additional discussion suggested by the reviewer in this revised manuscript. As mentioned by Reviewer 1, detailed mechanistic studies like applying the deuterated analog of DHED are out of the scope of this manuscript but they are currently underway in our laboratory and have been discussed in the revised manuscript. Please see the highlighted parts on pages 19, and 20 of the revised manuscript, which is as follows:**

"Our finding that DHEDs modulate ferroptosis-mediated neurodegeneration challenges the current paradigm in the field. Numerous studies demonstrated that membrane lipid composition and lipid peroxidation are essential for ferroptosis (Dixon et al., 2012; Ran et al., 2004; Wiernicki et al., 2020; W. S. Yang et al., 2016)**. Supplementation with PUFAs and their metabolites, particularly those metabolites with a higher degree of unsaturation, sensitizes cells to ferroptosis**(Dixon et al., 2012)**. However, unlike synthetic compounds such as Erastin, RSL-3, etc., these lipid molecules do not trigger ferroptosis, but rather act downstream of ferroptosis pathways by increasing the rate of membrane lipid peroxidation**(Dixon et al., 2012; Porter et al., 1981; Shintoku et al., 2017)**. This phenomenon is further supported by studies showing that supplementation with a monosaturated fatty acid, such as oleic acid, desensitizes cells from**

ferroptosis(Magtanong et al., 2019)**. In contrast, our results show that a specific ω-6 DGLA metabolite, DHED, induces ferroptosis, while other PUFAs (AA and EPA) and EPA metabolites (EEQ), with a higher degree of unsaturation, do not trigger ferroptosis. This observation aligns with a recent study showing that although EPA and AA supplementation are more deleterious in peroxide-induced whole-body oxidative stress, they cannot trigger ferroptotic germline cell death in *C. elegans***(Perez et al., 2022)**.**

Our data using Lip-1 supplementation, along with the use of transgenic strains carrying a loss of function *ftn-1* mutation, suggests that DHED could trigger lipid peroxidation in the ferroptosis pathway. However, it is unlikely that DHED induces ferroptosis-mediated neurodegeneration by undergoing peroxidation itself, as discussed above, because supplementation with AA, EPA, and EEQ, which are more prone to lipid peroxidation, have minimal or no effects in our neurodegenerative assays. In addition, it has been reported that dihydroxy-PUFAs are unable to incorporate into cell membranes(VanRollins et al., 1993)**, which suggests that DHEDs have a distinct mechanism for modulating ferroptosis compared to other PUFAs. It is because PUFAs with high degrees of unsaturation can propagate membrane lipid peroxidation during ferroptosis upon incorporation into the cell membrane. Although the exact mechanism underlying DHED induction of ferroptosis-mediated neurodegeneration is largely unknown, and falls beyond the scope of this study, we propose that DHED may interact with potential receptor proteins to activate the upstream ferroptosis pathway, leading to iron-mediated lipid peroxidation. This corroborates our finding from the experiments with Lip-1 and transgenic loss of function *ftn-1* strains, which indicate a critical role for lipid peroxidation in DHED-induced neurodegeneration. While DHED has not been extensively studied, similar metabolites, 9,10-dihydroxyoctadecenoic acid (DiHOME) and 12,13-DiHOME, which are dihydroxy-metabolites of LA, activate peroxisome proliferator-activated receptor (PPAR) gamma, and transient receptor potential vanilloid 1 (TRPV1), respectively**(Lecka-Czernik et al., 2002; Zimmer et al., 2018)**. In addition, 14,15-dihydroxyeicosatrienoic acid, dihydroxy-metabolites of AA, also activate PPAR alpha**(Fang et al., 2006)**. All of these proteins have been associated with ferroptosis**(Duan et al., 2022; Riegger, 2022; Venkatesh et al., 2020; Xing et al., 2022)**. Therefore, DHEDs could modulate ferroptosis-mediated neurodegeneration by interacting with one of these proteins or similar proteins. Alternatively, although DHED is less likely to be incorporated into the cell membrane, it could still be localized into specific subcellular compartments such as mitochondria, the endoplasmic reticulum (that contains the largest pool of lipids in cells), and lysosomes, where DHED could be peroxidized and propagate lipid peroxidation, leading to ferroptosis** (Dixon et al., 2014; Feng & Stockwell, 2018; Friedmann Angeli et al., 2014; Gan, 2021; Gaschler et al., 2018; Y. Yang et al., 2018)**. Currently, our laboratory is conducting a variety of genetic experiments to identify potential receptor proteins for DHED and synthesizing the deuterated DHED to investigate whether DHED peroxidation is necessary for their action in ferroptosis-mediated neurodegeneration."**

Reviewer 2:

Recent reports have linked lipid peroxidation and ferroptosis with the pathogenesis of neurodegeneration. A hallmark of ferroptosis is the accumulation of phospholipid hydroperoxides and polyunsaturated fatty acids are particularly prone to oxidative damage. In this manuscript, the authors explore the potential for PUFAs and PUFA metabolites to induce neurodegeneration using c. elegans as a tractable model system. Their results indicate that dihomo gamma linolenic acid (DGLA) is sufficient to trigger the degeneration of specific neurons via ferroptosis. They provide additional evidence that the DGLA metabolite, dihydroxyeicosadienoic acid (DHED), which is generated by cytochrome P450 (CYP) enzymes and epoxide hydrolases (EHs), mediates DGLA toxicity. They further suggest that the toxicity is not simply due to increased lipid peroxidation because the more highly peroxidizable EPA did not cause neurodegeneration.

Overall, this is an interesting study that is carefully performed, and the conclusions are wells supported. The concept that PUFA metabolites mediate cytotoxicity during ferroptosis and neurodegeneration is potentially important since it suggests a role for specific PUFA-derived metabolites and indicates PUFA metabolizing enzymes as therapeutic targets. What is perhaps lacking is a clear mechanism explaining how DGLA and DHED trigger ferroptosis.

Comment 1: The mechanism by which DHED triggers ferroptosis is not obvious and would benefit from discussion of potential models. The authors argue against the simple model that addition of PUFAs directly leads to an increase in lipid peroxidation. This seems plausible since EPA doesn't have the same effects (though it could be metabolized or absorbed differently) and there seems to be a high degree of specificity for DHED. Are these lipids incorporated into membranes? Do they impact membrane composition and / or lipid peroxidation defenses? Are they having other roles? MUFAs have been previously shown to suppress DGLA induced ferroptosis. This would suggest that membrane lipid composition and peroxidation is still essential for DGLA induced ferroptosis. A full mechanistic understanding is likely beyond the scope of this manuscript, but there should be additional discussion of the possible models of action.

Response: **We greatly appreciate the reviewer's positive feedback and insightful comments because they helped us to improve our manuscript and communicate our findings more effectively. A discussion on the potential mechanisms by which DHED could be involved in observed ferroptosis-induced neurodegeneration has been added to the revised version.**

Please kindly see the response to the reviewer 1, and the highlighted parts on page 19 to 20 of the revised manuscript.

Comment 2: It would be helpful to cite the original research articles for this statement rather than reviews – "… in contrast to conclusions reported previously,13,14,49 our data showed that treatment with the more peroxidizable arachidonic acid and EPA did not trigger neurodegeneration."

<u>Response:</u> Thank you very much for the suggestion. The original research articles are now cited. We also rephrased this statement to avoid any confusion.

The text on page 7 that addresses this comment is as follows:

"…In addition, while previous studies reported that the increased lipid peroxidation could induce neurodegeneration(Angelova et al., 2015; Fu et al., 2022; Galbusera et al., 2004; Praticò et al., 2001; Sultana et al., 2013)**, our data showed that the treatment of more peroxidizable arachidonic acid and EPA do not trigger neurodegeneration."**

<u>The following references were added to the revised version in order to respond to the reviewers' comments</u>

Angelova, P. R., Horrocks, M. H., Klenerman, D., Gandhi, S., Abramov, A. Y., & Shchepinov, M. S. (2015). Lipid peroxidation is essential for α-synuclein-induced cell death. *Journal of Neurochemistry*, *133*(4), 582–589.

Dixon, S. J., Lemberg, K. M., Lamprecht, M. R., Skouta, R., Zaitsev, E. M., Gleason, C. E., Patel, D. N., Bauer, A. J., Cantley, A. M., Yang, W. S., Morrison, B., 3rd, & Stockwell, B. R. (2012). Ferroptosis: an iron-dependent form of nonapoptotic cell death. *Cell*, *149*(5), 1060–1072.

Dixon, S. J., Patel, D. N., Welsch, M., Skouta, R., Lee, E. D., Hayano, M., Thomas, A. G., Gleason, C. E., Tatonetti, N. P., Slusher, B. S., & Stockwell, B. R. (2014). Pharmacological inhibition of cystine-glutamate exchange induces endoplasmic reticulum stress and ferroptosis. *ELife*, *3*, e02523.

Duan, C., Jiao, D., Wang, H., Wu, Q., Men, W., Yan, H., & Li, C. (2022). Activation of the PPARγ prevents ferroptosis-induced neuronal loss in response to intracerebral hemorrhage through synergistic actions with the Nrf2. *Frontiers in Pharmacology*, *13*, 869300.

Fang, X., Hu, S., Xu, B., Snyder, G. D., Harmon, S., Yao, J., Liu, Y., Sangras, B., Falck, J. R., Weintraub, N. L., & Spector, A. A. (2006). 14,15-Dihydroxyeicosatrienoic acid activates peroxisome proliferator-activated receptor-alpha. *American Journal of Physiology. Heart and Circulatory Physiology*, *290*(1), H55-63.

Feng, H., & Stockwell, B. R. (2018). Unsolved mysteries: How does lipid peroxidation cause ferroptosis? *PLoS Biology*, *16*(5), e2006203.

Friedmann Angeli, J. P., Schneider, M., Proneth, B., Tyurina, Y. Y., Tyurin, V. A., Hammond, V. J., Herbach, N., Aichler, M., Walch, A., Eggenhofer, E., Basavarajappa, D., Rådmark, O., Kobayashi, S., Seibt, T., Beck, H., Neff, F., Esposito, I., Wanke, R., Förster, H., … Conrad, M. (2014). Inactivation of the ferroptosis regulator Gpx4 triggers acute renal failure in mice. *Nature Cell Biology*, *16*(12), 1180–1191.

Fu, Y., He, Y., Phan, K., Bhatia, S., Pickford, R., Wu, P., Dzamko, N., Halliday, G. M., & Kim, W. S. (2022). Increased unsaturated lipids underlie lipid peroxidation in synucleinopathy brain. *Acta Neuropathologica Communications*, *10*(1), 165.

Galbusera, C., Facheris, M., Magni, F., Galimberti, G., Sala, G., Tremolada, L., Isella, V., Guerini, F. R., Appollonio, I., Galli-Kienle, M., & Ferrarese, C. (2004). Increased susceptibility to plasma lipid peroxidation in Alzheimer disease patients. *Current Alzheimer Research*, *1*(2), 103–109.

Gan, B. (2021). Mitochondrial regulation of ferroptosis. *The Journal of Cell Biology*, *220*(9). https://doi.org/10.1083/jcb.202105043

Gaschler, M. M., Hu, F., Feng, H., Linkermann, A., Min, W., & Stockwell, B. R. (2018). Determination of the subcellular localization and mechanism of action of ferrostatins in suppressing ferroptosis. *ACS Chemical Biology*, *13*(4), 1013–1020.

Lecka-Czernik, B., Moerman, E. J., Grant, D. F., Lehmann, J. M., Manolagas, S. C., & Jilka, R. L. (2002). Divergent effects of selective peroxisome proliferator-activated receptor-gamma 2 ligands on adipocyte versus osteoblast differentiation. *Endocrinology*, *143*(6), 2376–2384.

Magtanong, L., Ko, P.-J., To, M., Cao, J. Y., Forcina, G. C., Tarangelo, A., Ward, C. C., Cho, K., Patti, G. J., Nomura, D. K., Olzmann, J. A., & Dixon, S. J. (2019). Exogenous monounsaturated fatty acids promote a ferroptosis-resistant cell state. *Cell Chemical Biology*, *26*(3), 420-432.e9.

Perez, M. A., Clostio, A. J., Houston, I. R., Ruiz, J., Magtanong, L., Dixon, S. J., & Watts, J. L. (2022). Ether lipid deficiency disrupts lipid homeostasis leading to ferroptosis sensitivity. *PLoS Genetics*, *18*(9), e1010436.

Porter, N. A., Lehman, L. S., Weber, B. A., & Smith, K. J. (1981). Unified mechanism for polyunsaturated fatty acid autoxidation. Competition of peroxy radical hydrogen atom abstraction, .beta.-scission, and cyclization. *Journal of the American Chemical Society*, *103*(21), 6447–6455.

Praticò, D., Uryu, K., Leight, S., Trojanoswki, J. Q., & Lee, V. M. (2001). Increased lipid peroxidation precedes amyloid plaque formation in an animal model of Alzheimer amyloidosis. *The Journal of Neuroscience: The Official Journal of the Society for Neuroscience*, *21*(12), 4183–4187.

Ran, Q., Liang, H., Gu, M., Qi, W., Walter, C. A., Roberts, L. J., 2nd, Herman, B., Richardson, A., & Van Remmen, H. (2004). Transgenic mice overexpressing glutathione peroxidase 4 are protected against oxidative stress-induced apoptosis. *The Journal of Biological Chemistry*, *279*(53), 55137–55146.

Riegger, J. (2022). TRPV1 as an anti-ferroptotic target in osteoarthritis. *EBioMedicine*, *84*(104279), 104279.

Shintoku, R., Takigawa, Y., Yamada, K., Kubota, C., Yoshimoto, Y., Takeuchi, T., Koshiishi, I., & Torii, S. (2017). Lipoxygenase-mediated generation of lipid peroxides enhances ferroptosis induced by erastin and RSL3. *Cancer Science*, *108*(11), 2187–2194.

Sultana, R., Perluigi, M., & Butterfield, D. A. (2013). Lipid peroxidation triggers neurodegeneration: A redox proteomics view into the Alzheimer disease brain. *Free Radical Biology & Medicine*, *62*, 157–169.

VanRollins, M., Kaduce, T. L., Knapp, H. R., & Spector, A. A. (1993). 14,15-Epoxyeicosatrienoic acid metabolism in endothelial cells. *The Journal of Lipid Research*, *34*(11), 1931–1942.

Venkatesh, D., O'Brien, N. A., Zandkarimi, F., Tong, D. R., Stokes, M. E., Dunn, D. E., Kengmana, E. S., Aron, A. T., Klein, A. M., Csuka, J. M., Moon, S.-H., Conrad, M., Chang, C. J., Lo, D. C., D'Alessandro, A., Prives, C., & Stockwell, B. R. (2020). MDM2 and MDMX promote ferroptosis by PPARα-mediated lipid remodeling. *Genes & Development*, *34*(7–8), 526–543.

Wiernicki, B., Dubois, H., Tyurina, Y. Y., Hassannia, B., Bayir, H., Kagan, V. E., Vandenabeele, P., Wullaert, A., & Vanden Berghe, T. (2020). Excessive phospholipid peroxidation distinguishes ferroptosis from other cell death modes including pyroptosis. *Cell Death & Disease*, *11*(10), 922.

Xing, G., Meng, L., Cao, S., Liu, S., Wu, J., Li, Q., Huang, W., & Zhang, L. (2022). PPARα alleviates iron overload-induced ferroptosis in mouse liver. *EMBO Reports*, *23*(8), e52280.

Yang, W. S., Kim, K. J., Gaschler, M. M., Patel, M., Shchepinov, M. S., & Stockwell, B. R. (2016). Peroxidation of polyunsaturated fatty acids by lipoxygenases drives ferroptosis. *Proceedings of the National Academy of Sciences of the United States of America*, *113*(34), E4966-75.

Yang, Y., Lee, M., & Fairn, G. D. (2018). Phospholipid subcellular localization and dynamics. *The Journal of Biological Chemistry*, *293*(17), 6230–6240.

Zimmer, B., Angioni, C., Osthues, T., Toewe, A., Thomas, D., Pierre, S. C., Geisslinger, G., Scholich, K., & Sisignano, M. (2018). The oxidized linoleic acid metabolite 12,13-DiHOME mediates thermal hyperalgesia during inflammatory pain. *Biochimica et Biophysica Acta. Molecular and Cell Biology of Lipids*, *1863*(7), 669–678.
